# Neural Inefficiency During Dual-Task Walking in Older Adults: The Moderating Effect of Brain Morphology

**DOI:** 10.1093/geroni/igaa057.923

**Published:** 2020-12-16

**Authors:** Daliah Ross, Mark Wagshul, Meltem Izzetoglu, Roee Holtzer

**Affiliations:** 1 Yeshiva University, Bronx, New York, United States; 2 Albert Einstein College of Medicine, Bronx, New York, United States; 3 Villanova University, Villanova, Pennsylvania, United States

## Abstract

Aging is accompanied by changes in cognitive, physical, and brain function. Research has found increased activation in the prefrontal cortex (PFC) in dual-task-walk (DTW) as compared to single-task walk (STW) due to increased cognitive demands in the former condition. Older adults have more difficulty with DTW than younger adults, and difficulty with DTW has been found to predict functional decline in aging. As the population gets older, it is important to identify biomarkers predictive of functional decline. The neural inefficiency hypothesis posits that some people, including older adults, utilize more neural resources than others without showing benefits to behavioral performance. We examined the moderating effect of cortical thickness on PFC activation from STW to DTW. We predicted that less cortical thickness would be associated with increased PFC activation but without benefit to behavioral performance, to compensate for limited neural resources. Participants were 55 right-handed community-residing healthy older adults (mean age = 74.84 + 4.97; %female = 49.1). Cortical thickness was measured via MRI. Activation in the PFC was measured via functional near-infrared spectroscopy-derived (fNIRS) oxygenated hemoglobin (HbO2) levels. Analyses controlled for behavioral performance. Linear-mixed-effects models revealed that cortical thickness in several brain regions significantly moderated the increase in HbO2 from STW to DTW, p < 0.01. Thinner cortex, in regions implicated in walking and cognition, was associated with a greater increase in HbO2 levels. Supporting the neural inefficiency hypothesis, results suggest that older adults with greater cortical thinning over-activate the PFC during attention-demanding walking.

